# Cloning and constitutive expression of *Deschampsia antarctica *Cu/Zn superoxide dismutase in *Pichia pastoris*

**DOI:** 10.1186/1756-0500-2-207

**Published:** 2009-10-12

**Authors:** Jaime R Sánchez-Venegas, Alejandro Navarrete, Jorge Dinamarca, León A Bravo Ramírez, Ana Gutiérrez Moraga, Manuel Gidekel

**Affiliations:** 1 Laboratorio de Biología Molecular Aplicada, Instituto de Agroindustrias, Facultad de Ciencias Agropecuarias y Forestales, Universidad de La Frontera, Casilla 54-D, Temuco-Chile; 2 Departamento de Botánica, Facultad de Ciencias Naturales y Oceanográficas Universidad de Concepción, Casilla 160-C, Concepción-Chile; 3 VentureLab, Escuela de Negocios, Universidad Adolfo Ibáñez, Av. Diagonal Las Torres 2700, Peñalolén, Santiago-Chile

## Abstract

**Background:**

*Deschampsia antarctica *shows tolerance to extreme environmental factors such as low temperature, high light intensity and an increasing UV radiation as result of the Antarctic ozone layer thinning. It is very likely that the survival of this species is due to the expression of genes that enable it to tolerate high levels of oxidative stress. On that account, we planned to clone the *D. antarctica *Cu/ZnSOD gene into *Pichia pastoris *and to characterize the heterologous protein.

**Findings:**

The Copper/Zinc superoxide dismutase (Cu/ZnSOD) gene, *SOD *gene, was isolated from a *D. antarctica *by cDNA library screening. This *SOD *gene was cloned in the expression vector pGAPZαA and successfully integrated into the genome of the yeast *P. pastoris *SMD1168H. A constitutive expression system for the expression of the recombinant SOD protein was used. The recombinant protein was secreted into the YPD culture medium as a glycosylated protein with a 32 mg/l expression yield. The purified recombinant protein possesses a specific activity of 440 U/mg.

**Conclusion:**

*D. antarctica *Cu/ZnSOD recombinant protein was expressed in a constitutive system, and purified in a single step by means of an affinity column. The recombinant SOD was secreted to the culture medium as a glycoprotein, corresponding to approximately 13% of the total secreted protein. The recombinant protein Cu/ZnSOD maintains 60% of its activity after incubation at 40°C for 30 minutes and it is stable (80% of activity) between -20°C and 20°C. The recombinant SOD described in this study can be used in various biotechnological applications.

## Background

The hairgrass *Deschampsia antarctica *Desv. is one of the two vascular plants that have adapted to the extreme climate of the Maritime Antarctic [1]. It is tolerant to low temperatures, usually between -2°C and 6°C in summer and freezing in winter [2]. Episodes of high light intensity and increased UV radiation due to the thinning of the ozone layer during spring are frequent in Antarctic [3]. Because of the extreme conditions in which this plant lives, it has attracted the interest of plant physiologists, biochemists and molecular biologists, who are trying to discover the mechanisms that enable it to colonize the Antarctic environment [4-6].

It has been determined that the combination of low temperature, high light and UV radiation leads to an increase in the production of reactive oxygen species, such as the superoxide anion, hydrogen peroxide and the hydroxyl radical [7]. The accumulation of these reactive species in cells causes lipoperoxidation in membranes, DNA damage and the inactivation of enzymes [8]. The cell defense system, composed of enzymatic and non-enzymatic antioxidants, minimizes the deleterious effects of free radicals. SOD and other antioxidant enzymes such as catalase (CAT) and ascorbate peroxidase (APX) play a pivotal role detoxifying harmful reactive oxygen species. The SODs are classified into three types according to the metal they need as a co-factor: copper/zinc (Cu/ZnSOD), manganese (MnSOD) and iron (FeSOD) [9].

Taking into account the ability of *D. antarctica *to tolerate high levels of oxidative stress, it is highly probable that this plant expresses genes that allows it to survive the harsh Antarctic conditions such as the increase in ultraviolet radiation in Antarctic in recent decades, high light intensity and very low temperatures [2,4]. This assumption arises from the basis that tolerance to a wide variety of restrictive environmental factors is correlated to a greater activity of antioxidant enzymes and to the levels of antioxidant metabolites [5,6]. Plants with high levels of these antioxidative properties present tolerance to different and multiple types of environmental stresses.

The broad industrial application of SOD, such as use in cosmetics, cream and pharmaceutical compositions [10], have led investigators to clone SOD genes from different sources in order to express it in bacteria such as *Escherichia coli*. This has been done for the human MnSOD [11] and the Cu/ZnSOD of rice [12]. Other investigators prefer to use the methyltrophic yeast *Pichia pastoris *to express the SOD because of the advantages it offers when compared to other expression systems, such as a high growth rate at high-cell densities, using a simple culture medium. In addition, the recombinant protein can be exported to the culture medium, facilitating the protein recovery process. For these reasons, the yeast *P. pastoris *has been used in recent years to express many proteins with biotechnological perspectives [13]. Recombinant SOD expression in *P. pastoris *has been done for different SOD isozymes including the human SOD1 [14], the human extracellular SOD [15] and the SOD of *Sacchraromyces cervisiae *[16]. The motivation to study the gene that codes the Cu/ZnSOD of *D. antarctica *is based on the potential industrial applications which this protein could have, given the aforementioned peculiarities of this plant. Therefore, this report describes a system of constitutive expression in *P. pastoris *SMD1168H for the expression of the Cu/ZnSOD protein from *D. antarctica *and the biochemical properties of the recombinant protein.

## Methods

### Microorganism strains and growth conditions

*P. pastoris *SMD1168H (Invitrogen, USA) was grown at 28.5°C in either YPD or YEPD medium (Yeast Extract Peptone Dextrose Medium) and YPDS medium (YPD plus 1 M sorbitol), prepared according to instructions in the manual: EasySelect Echo-Adapted *Pichia *Expression kit of Invitrogen [17]. For growth on plates, 2% agar was added to the media. Transformed strains were cultivated in media supplemented with 100 μg/ml zeocin. For cloning procedures, *Escherichia coli *TOP 10F' (Invitrogen, USA) were used and grown at 37°C in LB medium supplemented with 25 μg/ml zeocin when required.

### DNA techniques

Molecular biology protocols were carried out according to Sambrook and Russell [18]. *E. coli *and *P. pastoris *cells were transformed by electroporation. Enzymes *Eco*RI, *Xba*I, *Avr*II and T4 DNA ligase (Fermentas, USA), *Taq *DNA polymerase and reagents for PCR (Invitrogen, USA) and PNGase F (NewEngland BioLab) were used as recommended by the supplier. DNA sequencing was performed by Macrogen (Korea). DNA sequences were analyzed using the Vector NTI Suite 7.0 (Invitrogen, USA).

### Isolation of the *D. antarctica SOD *gene

The *SOD *gene was isolated from a *D. antarctica *cDNA library, cloned into the pAGEN-1 vector (Agencourt). The screening, isolation and further sequence analysis is described in Sánchez-Venegas *et al *[19].

### Construction of the expression vectors

For the construction of the *SOD *expression vector *Eco*RI and *Xba*I sites were introduced respectively at the 5' and 3' end of *D. antarctica *Cu/ZnSOD by PCR with the primers pSOD-Fw (5'-AAGAATTCATGGTGAAGGCTGTAGCTGTGC-3') and pSOD-Rev (5'-ATATTCTAGACCCTGGAGCCCGATGATCC-3') using DNA from the *D. antarctica *cDNA library as template. The resulting 475 bp PCR product was cloned into the pGEM-T Easy vector (Promega, USA). The plasmid DNA was digested with *Eco*RI and *Xba*I and the *SOD *gene was cloned in frame with the α-factor secretion signal peptide at the N-terminal and with the myc epitope and the 6xHis-tag at the C-terminal into the pGAPZαA vector resulting in the pGAPZαA-SOD. The nucleotide sequence of the cloned PCR fragment was confirmed by sequencing (data not shown).

### *Pichia pastoris *transformation and selection

The pGAPZαA-SOD plasmid was linearized with *Avr*II in the GAP promoter region and transformed into *P. pastoris *SMD1168H electrocompetent cells. Zeocin resistant transformants were selected on YPDS agar plates. *P. pastoris *SMD1168H with empty vector pGAPZαA was transformed for negative control tests. Integration was confirmed by PCR following Linder *et al *amplification protocol [20] with the primers pGAP forward and 3'AOX1 reverse. Correct integration will result in the formation of a 1000 bp PCR product.

### Expression and purification of recombinant SOD

The selected clones' cultures (50 ml) were incubated for up to 96 hours at 28.5°C in a shaking incubator. Samples were taken every 24 hours for supernatant analysis. The cellular pellet was resuspended in 2× lysis buffer (100 mM tris-HCl pH: 8.0; 2 mM EDTA and 200 mM NaCl), containing PMSF at 1 mM final concentration. The cells lysis was performed by sonication (Sonicator Misonix, New York USA). The lysate was centrifuged at 14,000 rpm for 5 minutes at 4°C and the supernatant was kept at -80°C for further analysis. The culture medium supernatant (incubated for 96 h) was concentrated at 1/5th of its original volume before purification of recombinant SOD protein. This step was performed at 4°C by centrifugation at 3000 × g through a cellulose membrane with a pore diameter of 10 kDa (Centriplus YM-10, Millipore Corporation USA). The recombinant protein was purified by affinity chromatography using the ProBond Purification System (Invitrogen, USA). Native purification was carried out using a Ni-charged resin on poly-propylene cartridge. The concentrated sample was mixed with a volume of binding buffer (50 mM NaH_2_PO_4_, 50 mM Na_2_HPO_4_, and 500 mM NaCl, pH: 8.0) and agitated gently at 4°C for 1 hour. Four washes were made using wash buffer (binding buffer plus 50 mM Imidazole, pH 8.0). The SOD protein was eluted with 8 ml of elution buffer (binding buffer plus 500 mM Imidazole, pH 8.0) and aliquots of 1 ml of elution buffer were collected and stored at -80°C until further use.

### Recombinant protein characterization

Crude and purified samples were analyzed by SDS-PAGE 15%, stained with Coomassie Brillant Blue R-250 (Gibco BRL, USA) and by Western blot. The protein concentrations in the samples were determined with Bradford protein assay [21] using BSA as standard. For recombinant protein immunodetection the IgG anti-myc specific antibody (Invitrogen, USA) was used and developed using the chromogene method that utilizes the secondary antibody Goat anti-mouse IgG Alkaline Phosphatase conjugate (Upstate, USA). Recombinant Cu/ZnSOD was immunodetected with primary antibody IgG anti-Cu/ZnSOD (Envirtue Biotechnologies) and was developed via the chemiluminescent method (GE Healthcare, UK). Bovine erythrocytes Superoxide dismutase (Calbiochem, Merck Germany) was used as control.

Zymograms were prepared according to Rao *et al*. [22] and Chen and Pan [23]. The proteins were separated by PAGE under non denaturing conditions. In order to visualize the enzyme bands, the native gel was incubated in a 2.5 mM nitroblue tetrazolium solution for 25 minutes. Then, the gels were incubated in 50 mM sodium phosphate buffer (pH: 7.8) containing 28 μM riboflavin and 28 mM tetramethyl-ethylene-diamine for 20 minutes in darkness. After placing the gels in water, the zymograms were developed by exposure to light at room temperature. White bands on a dark-blue background indicate the presence of SOD activity.

The spectrophotometry activity of recombinant SOD was determined following the protocol of McCord and Fridovich [24], with modifications by Schöner and Krause [25]. The activity was assayed determining the proportion of ferricytochrome C reduced at 550 nm in a thermoregulated spectrophotometer. One unit of SOD is defined as the amount of enzyme required to inhibit the reduction of ferricytochrome C by 50% at pH 7.8 and 25°C. The thermostability of the purified SOD enzyme was determined by measuring its activity at different temperatures. The recombinant protein samples were incubated for 30 minutes at temperatures of -20, 0, 20, 40, 60, 80 and 100°C. For below zero temperatures (-20°C) glycerol 50% was added. Enzyme activity after autoclaving at a pressure of 1 atmosphere for 20 minutes was also determined. The results of these experiments were expressed as a percentage of activity against the activity measured at 25°C.

For deglycosylation, crude and purified samples were treated with "Peptide N-Glycosydase F" (PNGase F) following the supplier's instructions (New England BioLab). The results of the deglycosylation and respective controls without PNGase F treatment were visualized through SDS-PAGE gel (15%), stained with Coomassie Brillant Blue R-250 and Western blot. The blot was immunodecorated with IgG anti-myc antibody.

## Results and Discussion

### Cu/ZnSOD gene of *D. Antarctica*

**Figure 1:**
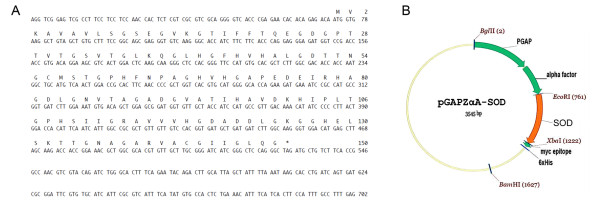
**Nucleotide sequence of clone C13 of the cDNAs library of *D. antarctica *and plasmid construct**. **A**. The deduced amino acid sequence is shown in one-letter code above the nucleotide sequence. Asterisks indicate the stop signal. **B**. Recombinant plasmid vector. The *SOD *gene from *D. antarctica *was cloned in frame with the α-factor secretion signal peptide at the N-terminal and with the myc epitope and the 6xHis-tag at the C-terminal into the pGAPZαA vector resulting in the pGAPZαA-SOD.

The clone C13 from *D. antarctica *cDNA library was identified as a Cu/ZnSOD and was selected for expression in *P. pastoris*. This *SOD *gene has an ORF of 456 bp encoding a protein of 152 amino acids with a molecular mass of approximately 15 kDa (Figure 1A). The *SOD *gene sequence shows high homology with Cu/ZnSOD genes from *Oryza sativa *and *Zea mays *[19].

### Expression of recombinant SOD in *Pichia pastoris*

The SOD coding sequence from *D. antarctica *(*SOD*) was cloned downstream from the α-factor signal sequence into the vector pGAPZαA, resulting in the pGAPZαA-SOD expression plasmid (Figure 1B). The pGAPZαA-SOD sequence was integrated to the *P. pastoris *SMD1168H genome in order to express the heterologous Cu/ZnSOD protein. The transformation of *P. pastoris *with the vector pGAPZαA-SOD was confirmed by amplification of the expression cassette by PCR using the primers PGAP forward and 3'AOX1 reverse. The amplification product is a DNA fragment of approximately 1000 bp (data not shown). The transformed clone SMD1168H-pGAPZαA-SOD and the control strain SMD1168H transformed with the empty vector were incubated in YPD medium for 96 hours at 28.5°C under constant agitation (250 rpm).

**Figure 2:**
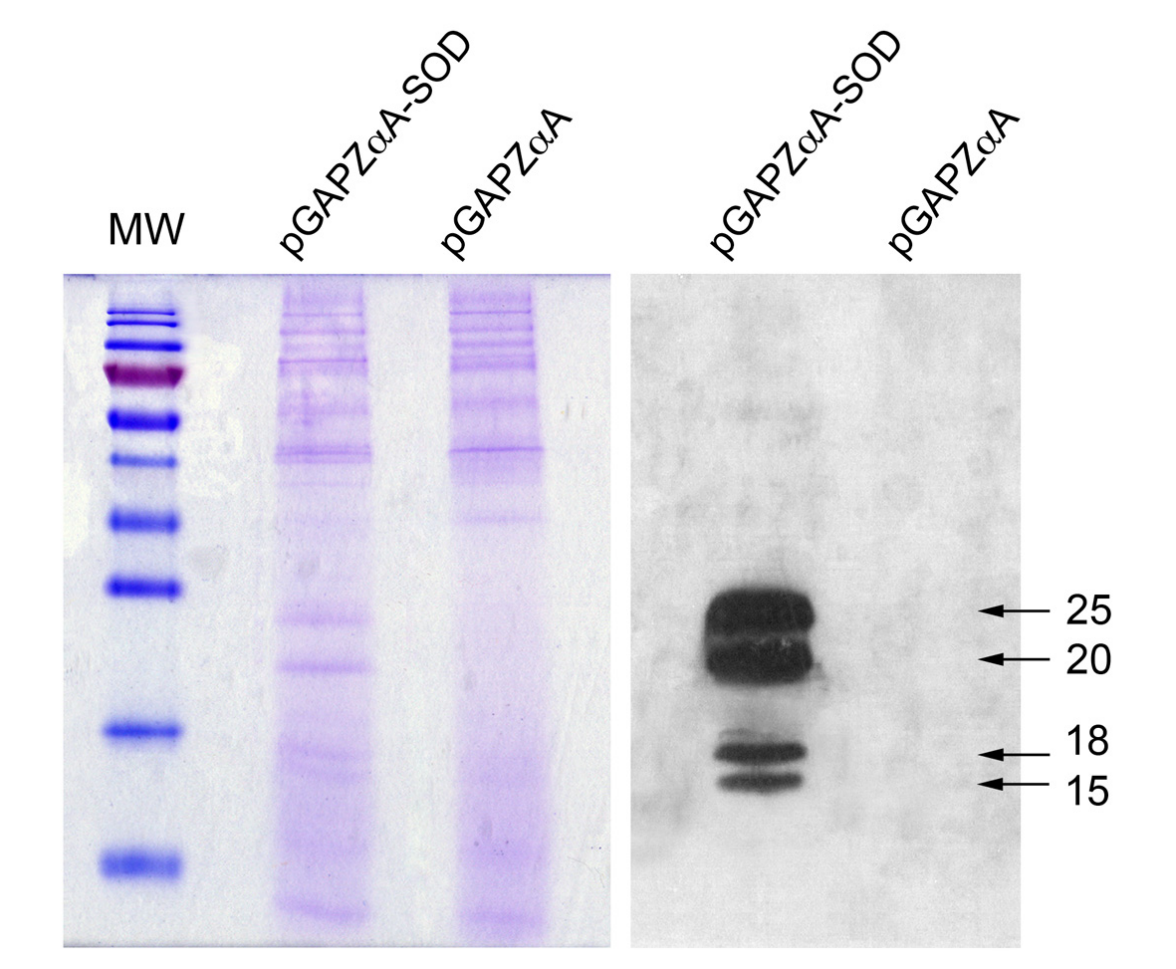
**Determination of recombinant Cu/ZnSOD protein expressed by *P. pastoris *SMD1168H**. Concentrated supernatant samples (6-fold) of the yeast culture incubated for 96 hours at 28.5°C were analyzed by 15% SDS-PAGE staining (left). Immunoblots were immunodecorated with Cu/ZnSOD specific antisera (right). Mw, molecular weight marker (Fermentas); pGAPZαA-SOD, supernatant of SMD1168H-pGAPZαA-SOD; pGAPZαA, supernatant of SMD1168H transformed with the empty vector (background expression). The arrows indicate bands of the secreted recombinant SOD protein.

**Figure 3:**
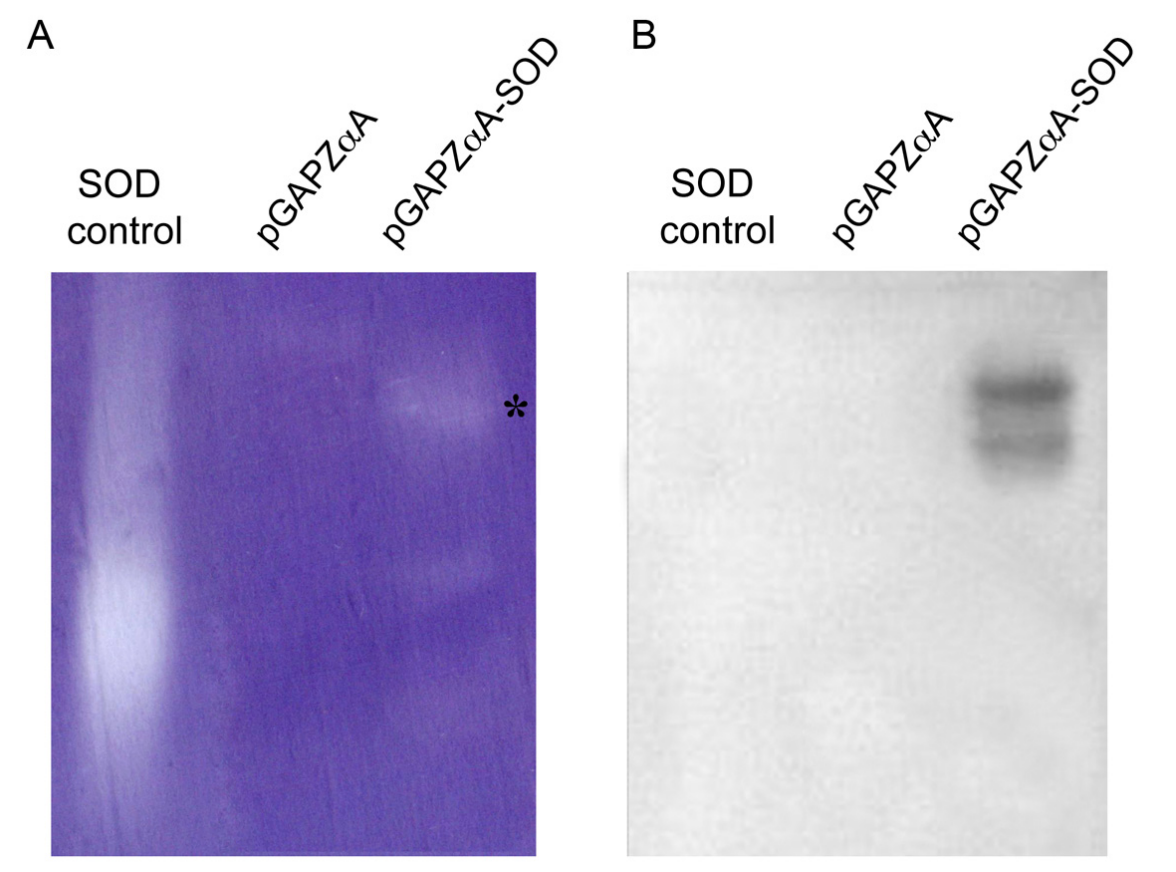
**Determination of enzymatic activity by zymogram and immunoblotting**. Supernatant samples from *P. pastoris *SMD1168H transformants pGAPZαA (background expression) and pGAPZαA-SOD were collected after incubation for 96 hours at 28.5°C. As a positive control, 3 U of SOD were used. **A**. SOD activity under non-denaturing conditions. Concentrated protein samples (6×) were separated in native-PAGE 12%. SOD activity is visualized as white bands (asterisk). **B**. Protein samples (1×) were analyzed by immunoblotting using myc-specific (Myc) antisera.

SDS-PAGE and Western blot were conducted to determine if the recombinant protein secreted by the transformant strain SMD1168H-pGAPZαA-SOD correspond to the *D. antarctica *Cu/ZnSOD (Figure 2). The immunodetection shows two bands of 25 and 20 kDa that correspond to the mature Cu/ZnSOD fused with the epitope-myc and 6xHis-tag and two additional bands of 18 and 15 kDa. These lower molecular weight proteins may correspond to immature proteins or proteins where the epitope-myc and 6xHis-tag has been lost as result of cellular damage (Figure 2). The immunodetection of supernatant samples with the anti-myc antibody shows that the mature recombinant proteins are secreted to the culture medium (Figure 3B). Additionaly, the 25 and 20 kDa proteins band were not detected in the Western blot analysis of celular lysates from the SMD1168H-pGAPZαA-SOD and SMD1168H strains using the anti-myc antybody (data not shown).

The recombinant SOD protein secreted into the culture medium corresponds to 13% of the total extracellular protein secreted by *P. pastoris*. Expression yields of different SOD proteins by other research groups are similar to our results [13-15]. It must be considered that the expression yields are not identical for all SOD genes, due to multiple factors, such as: source of the gene which presents variability in its sequence; restriction enzyme used to generate the linearized DNA of the expression vector, which affects insertion in the host genome; host type used to make the expression, among others.

### Activity determination of recombinant SOD secreted by *Pichia pastoris*

The activity of the recombinant SOD secreted by SMD1168H-pGAPZαA-SOD was measured by means of a spectrophotometric assay. The highest activity of the *SOD *was detected after 72 hours of transformed *P. pastoris *growth (data not shown). Low activity levels were detected in samples secreted by the SMD1168H strain that most likely correspond to the host SOD (data not shown). Recombinant SOD activity was determined in zymograms and by means of Western blot analysis (Figure 3). The zymogram assay shows a positive reaction when using a 6× concentrated sample (Figure 3A). The higher sensitivity of the immunodetection allows us to detect the recombinant SOD protein in the non-concentrated 1× samples (Figure 3B).

### Purification of recombinant SOD expressed by *Pichia pastoris*

The culture medium supernatant was concentrated previously to the purification of recombinant SOD protein as described in Methods. The purification of the SOD secreted by SMD1168H-pGAPZαA-SOD was established in a one-step procedure using a nickel affinity column (Probond Purification System, Invitrogen). The initial quantity and specific activity of the recombinant SOD in the culture supernatant was 11.25 mg and 80 U/mg, respectively. After purification, we obtained 0.256 mg of SOD protein with a specific activity of 440 U/mg and a total activity of 112.64 U. The purification results are summarized in Table 1. The SDS-PAGE and Western blot using the purified protein samples showed the same bands of 20 and 25 kDa as the non-purified samples. Despite the low purification yield (12.5), the use of this method allows a fast and less cumbersome purification compared to other methods such as the traditional method by precipitation with ammonium sulphate and dialysis. Moreover, the obtained amount of protein was sufficient for the characterization of the recombinant SOD.

**Table 1:** Purification of recombinant SOD expressed by *Pichia pastoris*

**Step**	**Volume** **(ml)**	**Protein** **(mg)**	**Total activity** **(U)**	**Specific activity** **(U/mg)**	**Yield** **(%)**
Supernatant	45	11.25	900	80	100.0
Concentrated (1/5)	9	6.40	134	21	14.9
ProBond	8	0.26	113	440	12.5

### Deglycosylation and thermostability of recombinant SOD

**Figure 4:**
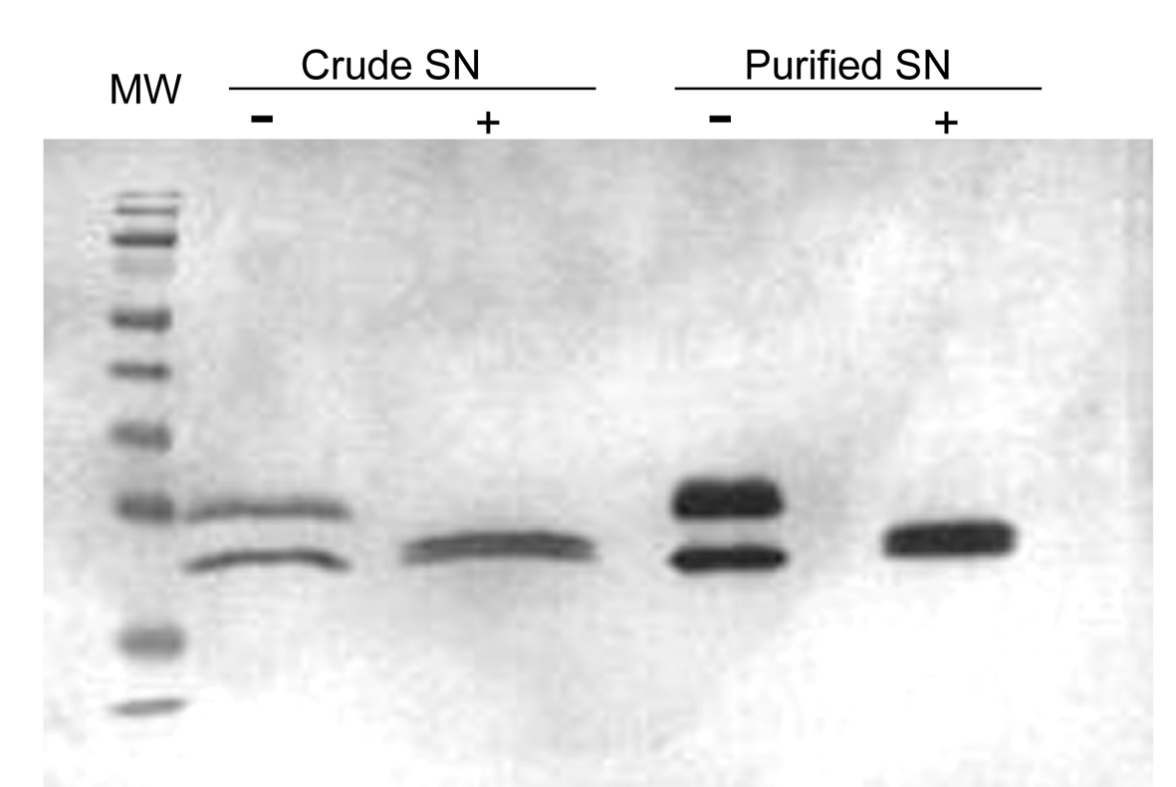
**Deglycosylation of recombinant SOD by treatment with PNGase F**. Crude and purified supernatant (SN) samples from the SMD1168H-pGAPZαA-SOD strain were treated without (-) or with (+) 1000 U PNGase F. After the deglycosylation treatment the samples were analyzed by SDS-PAGE and immunoblotting using myc-specific (Myc) antisera. Mw, molecular weight marker (Fermentas).

The recombinant SOD shows two separate bands of approximately 25 and 20 kDa on both native and SDS-PAGE (Figures 2, 3). These results suggest to us that the recombinant SOD could be secreted as a glycosylated protein. In order to verify this hypothesis, a deglycosylation test was performed. The deglycosylation assays with pNGase F on both crude and purified recombinant SOD were analyzed by SDS-PAGE and Western blot. The results showed two close bands of approximately 20 kDa in samples treated with pNGase F with the disappearance of the 25 kDa band (Figure 4). The 20 kDa proteins correspond to the molecular mass inferred by the nucleotide sequence cloned in frame with the myc epitope and the 6xHis-tag of pGAPZαA. Therefore, these results suggest that the strain SMD1168H-pGAPZαA-SOD is secreting the recombinant SOD as a glycoprotein. In plants, two classes of Cu/ZnSOD have been described. The first corresponds to the cytoplasmic homodimeric form, while the second class includes the chloroplastic and extracellular homotetrameric form; In addition, the homodimeric form has been detected in the periplasm of procariotic cells [26]. Glycosylated SODs are found in mammals and humans i.e. the intracellular SOD (Cu/ZnSOD), a homodimer with a molecular weight of 32 kDa, and whose monomers have a molecular mass of 15-18 kDa [27].

**Figure 5:**
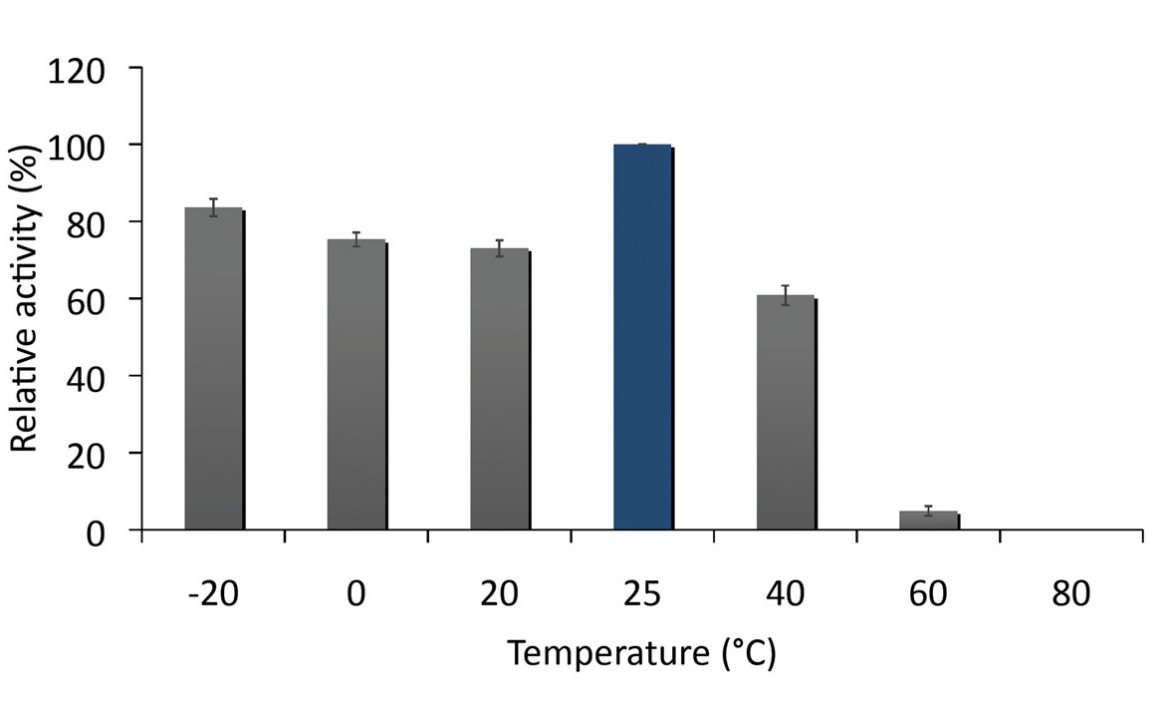
**Thermostability of recombinant SOD**. The activity of recombinant *SOD *was measured by spectrophotometry (see Methods). Previous to the activity measurement, samples were incubated at -20, 0, 20, 25, 40, 60 and 80°C for 20 minutes. The activity is expressed as the percentage of activity compared to the untreated sample at 25°C.

The thermostability assays of purified recombinant SOD was performed at different temperatures by means of the spectrophotometric assay. The results shows that after incubation for 30 minutes at 40°C the activity decrease by 40% and after 30 min at 60°C only 5% of the activity measured at 25°C remains (Figure 5). The assays performed above 60°C and after autoclave did not show enzymatic activity (data not shown). Similar studies performed on Cu/ZnSOD from *Oryza sativa *and *Citrullus vulgaris*, plants that are not adapted to low temperature environments, showed higher thermostability at temperatures over 60°C [12,28]. Thus, the relatively moderated thermostability at temperatures over 40°C and the apparent stability at temperatures between -20°C and 20°C suggest that the Cu/ZnSOD from *D. antarctica *is adapted to low temperatures.

## Conclusion

The effectiveness of *P. pastoris *SMD1168H for obtaining Cu/ZnSOD has been demonstrated using a constitutive expression system reaching its highest expression after 96 hours, without the need for any inductor or carbon source exchange for switching on the expression of the foreign gene. The recombinant SOD protein is secreted to the culture medium as a glycoprotein, and corresponds to approximately 13% of the total protein secreted by the SMD1168H-pGAPZαA-SOD. The secretion of the recombinant protein to the culture medium facilitates concentration by simple filtration and purification in a two step procedure.

The recombinant SOD protein maintain 60% of its activity after incubation at 40°C for 30 minutes, however at temperatures above 60°C its activity is completely lost. On the other hand, the recombinant SOD maintains a rather stable activity (80% approximately) from -20°C and 20°C.

The recombinant SOD protein described could be used in diverse applications previously described for other SODs, such as the formulation of cosmetics and pharmaceutical creams that detain the aging process and provide photoprotection against UV radiation [29,30]; therapies to treat diseases in which Cu/ZnSOD plays an important role, such as rheumatoid arthritis, inflammatory diseases and neurodegenerative disorders [10].

## Competing interests

The authors declare that they have no competing interests.

## Authors' contributions

JRSV: cloned the gene, performed experiments of molecular characterisation and gene expression and wrote the manuscript. AN: participated in the experiments of activity and thermostability. JD: reviewed and corrected the manuscript. LABR: designed and conducted experiments of activity and thermostability. AG: constructed the *D. antarctica *cDNA library. MG: participated in the coordination of the project. All authors read and approved the final manuscript.

## References

[B1] Alberdi M, Bravo LA, Gutiérrez A, Gidekel M, Corcuera LJ (2002). Ecophysiology of antarctic vascular plants. Physiol Plant.

[B2] Bravo LA, Ulloa N, Zuñiga G, Casanova A, Corcuera LJ, Alberdi M (2001). Cold resistance in antarctic angiosperms. Physiol Plant.

[B3] Karentz D, Huiskes AHL, Gieskes WWC, Rozema J, Schorno RML, vander Vies SM, Wolff WJ (2003). Environmental change in Antarctica: ecological impacts and responses. Antarctic biology in a global context.

[B4] Gidekel M, Destefano-Beltran L, Garcia P, Mujica L, Leal P, Cuba M, Fuentes L, Bravo LA, Corchera LJ, Alberdi M, Concha I, Gutiérrez A (2003). Identification and characterization of three novel cold acclimation-responsive genes from the extremophile hair grass *Deschampsia antarctica *Desv. Extremophiles.

[B5] Pérez-Torres E, García A, Dinamarca J, Alberdi M, Gutiérrez A, Gidekel M, Ivanov A, Huner N, Corcuera L, Bravo L (2004). The role photochemical quenching and antioxidants in photoprotection of *Deschampsia antarctica*. Funct Plant Biol.

[B6] Pérez-Torres E, Bravo LA, Corchera LJ, Jonson GN (2007). Is electrón transport to oxygen an important mechanism in photoprotection? Contrasting responses from Antarctic vascular plants. Physiol Plant.

[B7] Prasad TK, Anderson MD, Martin BA, Stewart CR (1994). Evidence for chilling-induced oxidative stress in maize seedling and a regulatory role for hydrogen peroxide. Plant Cell.

[B8] Blokhina O, Virolainen E, Fagerstedt KV (2003). Antioxidant, oxidative damage and oxygen deprivation stress: a review. Annals of Botany.

[B9] Scandalios JG (2005). Oxidative stress: molecular perception and transduction of signal triggering antioxidant gene defenses. Braz J Med Biol Res.

[B10] Ratnam DV, Ankola DD, Bhardwaj V, Sahana DK, Kumar MNVR (2006). Role of antioxidants in profhylaxis and therapy: a pharmaceutical perspective. J Control Release.

[B11] Beck Y, Bartfeld D, Yavin Z, Levanon A, Gorecki M, Hartman JR (1988). Efficient production of active human manganese superoxide dismutase in *Escherichia coli*. Nat Biotechnol.

[B12] Pan SM, Hawang GB, Liu HC (1999). Over-expression and characterization of copper/zinc-superoxide dismutase from rice in *Escherichia coli*. Bol Bull Acad Sci.

[B13] Cregg JM, Vedvick TS, Raschke WC (1993). Recent advances in the expression of foreign genes in *Pichia pastoris*. Biotechnology.

[B14] Yoo HY, Kim SS, Rho HM (1999). Overexpression and simple purification of human superoxide dismutase (SOD1) in yeast and its resistance to oxidative stress. J Biotechnol.

[B15] Chen HL, Yen CC, Tsai TC, Yu CH, Liou YJ, Lai YW, Wang ML, Chen CM (2006). Production and characterization of human extracellular superoxide dismutase in the methylotrophic yeast *Pichia pastoris*. J Agric Food Chem.

[B16] Yu P (2007). A new approach to the production of the recombinant SOD protein by methylotrophic *Pichia pastoris*. Appl Microbiol Biotechnol.

[B17] Invitrogen Pichia expression vectors for constitutive expression and purification of recombinant proteins. Catalog N° V200-20 and V205-20.

[B18] Sambrook J, Russell DW (2001). Molecular cloning a laboratory manual.

[B19] Sánchez-Venegas JR, Dinamarca J, Gutiérrez A, Gidekel M (2009). Molecular characterization of a cDNA encoding Cu/Zn superoxide dismutase from *Deschampsia antarctica *and its expression regulated by cold and UV stresses. BMC Res Notes.

[B20] Linder S, Schliwa M, kube-Granderath E (1996). Direct PCR Screening of *Pichia pastoris *Clones. Biotechniques.

[B21] Bradford MM (1976). A rapid and sensitive method for the quantitation of microgram quantities of protein utilizing the principle of protein-dye binding. Anal Biochem.

[B22] Rao MV, Paliyath G, Ormrod DP (1996). Ultraviolet-b- and ozone-induced biochemical changes in antioxidant enzymes of *Arabidopsis thaliana*. Plant Physiol.

[B23] Chen CN, Pan SM (1996). Assay of superoxide dismutase activity by combining electrophoresis and densitometry. Bot Bull Acad Sinica.

[B24] McCord JM, Fridovich I (1969). Superoxide dismutase. An enzymic function for erythrocuprin (hemocuprin). J Biol Chem.

[B25] Schöner S, Krause GH (1990). Protective systems against active oxygen species in spinach: response to cold acclimation in excess light. Planta.

[B26] Bordo D, Djinovic K, Bolognesi M (1994). Conserved patterns in the Cu, Zn superoxide dismutase family. J Mol Biol.

[B27] Fridovich I (1975). Superoxide dismutase. Annu Rev Biochem.

[B28] Bueno P, Varela J, Ciménez-Gallego G, del Rio LA (1995). Peroxisomal Copper, Zinc Superoxide Dismutase. Characterization of the lsoenzyme from Watermelon Cotyledons. Plant Physiol.

[B29] Scharffetter-Kochanek K, Wlaschek M, Brenneisen P, Schauen M, Blaudschun R, Wenk J (1997). UV-induced reactive oxygen species in photocarcinogenesis and photoaging. Biol Chem.

[B30] Takahashi H, Hashimoto Y, Aoki N, kinouchi M, Ishida-Yamamoto A, Iizuka H (2000). Copper, zinc-superoxide dismutase protects from ultraviolet B-induced apoptosis of SV40-transformed human keratinocytes: the protection is associated with the increased levels of antioxidant enzymes. J Dermatol Sci.

